# Focal volume, steering, and aberration correction in transcranial focused ultrasound: reconsidering the tradeoffs between single-element transducers, phased arrays, and acoustic holograms

**DOI:** 10.3389/fnins.2026.1891259

**Published:** 2026-07-03

**Authors:** Norman M. Spivak, Andrew A. E. D. Bishay, Amber R. Hopkins, Bradley S. Bohall, Mark E. Schafer, Susan Y. Bookheimer, Martin M. Monti, Alexander Bystritsky

**Affiliations:** 1 Department of Psychiatry & Biobehavioral Sciences, UCLA, Los Angeles, CA, United States; 2 Department of Neurosurgery, UCLA, Los Angeles, CA, United States; 3 UCLA-Caltech Medical Scientist Training Program, Los Angeles, CA, United States; 4 Department of Psychology, UCLA, Los Angeles, CA, United States; 5 Rockefeller Neuroscience Institute, West Virginia University, Morgantown, WV, United States; 6 School of Biomedical Engineering, Science and Health Systems, Drexel University, Philadelphia, PA, United States

**Keywords:** electronic steering, focused ultrasound, phase correction, skull aberration correction, transducers

## Abstract

Transducer architecture is an increasingly important design consideration in transcranial focused ultrasound, where focal precision, electronic steering, skull-aberration correction, workflow complexity, and cost must be balanced. Under matched aperture, transmit frequency, focal depth, effective source geometry, and equivalent aberration correction, subdividing an aperture into multiple independently driven elements does not by itself reduce the diffraction-limited focal volume. Phased arrays provide important capabilities that single-element transducers generally lack, including electronic steering, dynamic phase correction, and flexible control of energy deposition. These capabilities can improve the realized intracranial field, particularly when skull-induced aberrations would otherwise broaden, shift, or split the focus. Single-element systems may also be extended through patient-specific acoustic holograms or lenses, which impose spatially varying phase delays to compensate for aberrations, steer the focus, or shape the pressure field. These approaches may reduce system complexity but are typically static and less adaptable than phased arrays. We therefore frame transducer selection as an application-driven design-matching problem: the relevant question is not which architecture is inherently “more focused” under matched conditions, but which best meets a study’s requirements for steering, skull-aberration correction, temporal reconfigurability, spatial degrees of freedom, workflow burden, cost, positioning robustness, and safety.

## Introduction

Focused ultrasound relies on precise energy delivery, making focal size a critical factor in neuromodulation, ablation, drug delivery, and other therapeutic applications. However, focal volume should be distinguished from other capabilities such as electronic steering, aberration correction, and dynamic control of energy deposition. In idealized free-field conditions, or when aberrations are equivalently corrected, the focal dimensions of an ultrasound source are governed primarily by wavelength, aperture, focal depth, and effective source geometry (O’Neil, 1949; Kossoff, 1979). For a given frequency and focal distance, increasing the effective aperture generally narrows the focus, whereas deeper focusing and longer wavelengths generally broaden it. Subdividing the same aperture into multiple independently driven elements does not, by itself, overcome these diffraction constraints. Thus, the relevant question is not whether a multi-element array is categorically “more focused” than a single-element transducer. Instead, the primary consideration should be the additional capabilities the array provides within the anatomical and operational constraints of a given application. For neuroscience applications, this distinction is best understood through an application-centered lens: different transducer architectures should be evaluated not only by idealized focal metrics but also by how well they match the operational demands of a specific targeting and study-design scenario.

### Capabilities and tradeoffs of phased arrays

Multi-element phased arrays provide two major capabilities beyond fixed geometric focusing: electronic steering and element-wise aberration correction. Electronic steering allows the focus to be shifted without mechanically repositioning the transducer, while element-wise phase and amplitude control can compensate for propagation-induced phase errors, including those caused by the skull. These capabilities can improve the realized intracranial field even when they do not reduce the idealized diffraction-limited focal volume. In other words, phased arrays may produce a smaller, cleaner, or more accurately located focus than an uncorrected single-element transducer in transcranial applications because of their ability to restore constructive interference at the intended target.

The benefits of phased arrays are accompanied by design tradeoffs. As the focus is electronically steered away from the array’s natural geometric focus, focal quality can degrade: the main lobe may widen, peak pressure may fall, and sidelobes or grating lobes may become more prominent, with the extent of these effects depending on steering angle, wavelength, element size, inter-element spacing, and element directivity. The effects are not inevitable in all designs; appropriate element sizing and spacing can expand the usable steering range and reduce or avoid grating lobes. However, such improvements typically require more elements, more independent channels, tighter calibration, and greater hardware and software complexity. Therefore, array performance depends both on whether a transducer is multi-element and on how the aperture is sampled, driven, calibrated, and integrated into the treatment-planning workflow.

The focal volume directly impacts treatment specificity in therapeutic applications such as high-intensity focused ultrasound (HIFU) for tissue ablation and lower-intensity applications for neuromodulation. A larger-than-expected or poorly localized focus can increase off-target exposure, reduce anatomical specificity, alter the interpretation of physiological or behavioral effects, and, in sufficiently high-energy applications, may increase safety risk (Kyriakou et al., 2014). However, the clinically realized focus is determined by both source geometry and propagation through intervening tissue. In homogeneous media, matched-aperture single-element and multi-element sources are subject to the same diffraction constraints. In heterogeneous media, particularly in transcranial applications, aberration correction can determine whether the intended focus is preserved, displaced, broadened, or split (Leung et al., 2021). This distinction is central: Phased arrays do not inherently reduce the diffraction-limited focal volume; however, they can improve the realized focal field when employed to correct aberrations or regulate off-target energy deposition.

### Transcranial aberration correction and treatment planning

Transcranial applications illustrate why this distinction matters. The skull introduces attenuation, refraction, reflection, and phase aberrations that can substantially degrade the intracranial field. With a phased array, patient-specific phase corrections can be applied across individual elements so that the transmitted waves interfere constructively at the target (Hynynen et al., 2004). This capability can preserve or restore focal quality in situations where an uncorrected single-element transducer would produce a broadened, shifted, or fragmented focus. Nevertheless, array-based aberration correction introduces practical complexity. Treatment planning may require patient-specific imaging, acoustic modeling, image-to-transducer registration, element-wise calibration, knowledge of the source output, and integration of hardware and software control pipelines. CT-based approaches remain common, while MR-derived synthetic CT methods and faster propagation models may reduce some of the imaging and computational burden (Kyriakou et al., 2014; Leung et al., 2021; Wang et al., 2025; Leung et al., 2022; Koh et al., 2022). Thus, phased arrays provide powerful correction capabilities, but they entail workflow, cost, and implementation requirements that may not be necessary for all applications. Separate from aberration correction, emerging planning tools that use individualized anatomical models may help optimize transducer placement and targeting geometry. These approaches are useful for prospective planning but must be distinguished from acoustic propagation models that explicitly estimate phase aberration, attenuation, and focal distortion through the skull (Lueckel et al., 2025).

### Single-element systems with acoustic holograms and lenses

Single-element systems can also be extended through patient-specific acoustic holograms or lenses (Attali et al., 2025; Maimbourg et al., 2020; Maimbourg et al., 2018). Rather than dynamically adjusting the phase of individual array elements, these devices impose a designed spatial phase distribution within the coupling path. By varying lens thickness, material properties, or geometry, an acoustic hologram can introduce spatially varying phase delays that compensate for skull-induced aberrations, steer the focus, or generate prescribed focal patterns. In this framework, the hologram does not function by matching the skull’s curvature or creating a generally “smoother” acoustic pathway; it transforms the incident acoustic field so that the transmitted field better matches the desired pressure distribution at the target.

Hologram-based approaches, therefore, offer a potential middle ground between simple single-element focusing and fully programmable phased arrays. They may reduce the need for large numbers of independent electronic channels and may be attractive for lower-cost or more portable systems. However, this simplicity comes at the expense of flexibility. A static hologram is typically designed for a particular transducer position, target, coupling configuration, and patient anatomy. If the target changes, the transducer is repositioned, or the treatment plan requires rapid steering across multiple sites, a new hologram or compromise design may be required. In contrast, a phased array can update phase delays electronically and can therefore support dynamic steering, retargeting, and adaptive correction more readily than a static lens.

## Discussion

To make these tradeoffs practical, Table 1 summarizes how representative transcranial ultrasound neuromodulation scenarios map onto key design requirements and indicates when a single-element transducer, a hologram- or lens-assisted system, or a phased array is likely to be the most appropriate architecture. These considerations suggest that transducer selection should be driven by multiple variables, and not solely by effective intracranial focal volume. A single-element transducer may be appropriate when the target is fixed, the required steering range is small, aberration is modest or acceptable, and system simplicity is prioritized. A single-element transducer with a patient-specific acoustic hologram may be appropriate when a static correction can compensate for skull-induced phase delays or produce a desired field pattern without the cost and complexity of a multi-channel array. A phased array may be preferred when the application requires dynamic steering, multi-target protocols, adaptive aberration correction, or flexible control over energy deposition. The optimal system, therefore, depends on the target, skull path, required accuracy, acceptable workflow burden, and clinical or experimental context.

**Table 1:** Qualitative decision aid for matching transcranial ultrasound architecture to study requirements.

Representative scenario	Targeting mode	Highest-priority requirements	Favored architecture	Main drawback
Repeated stimulation of one fixed cortical or moderately deep target	Fixed	Low workflow burden, low cost, straightforward setup, limited need for retargeting	Single-element transducer	Least flexible if the target or skull path changes
Repeated stimulation of one fixed deep or skull-challenging target with individualized correction	Fixed after upfront planning	High realized focal quality after skull propagation, subject-correction	Single-element transducer + patient-specific hologram/lens	Correction is typically static and depends on stable geometry and positioning
Rapid switching among cortical and subcortical targets in the same session	Dynamic	Electronic steering, rapid retargeting, on-the-fly phase updates, high temporal reconfigurability	Phased array	Higher hardware, calibration, and planning complexity
Fixed multifocal or structure-conforming stimulation pattern	Fixed patterned field	Multiple simultaneous foci or target-conforming field shaping without dynamic updates	Hologram/lens- assisted system	Well suited to fixed patterns, but not to frequent pattern changes
Multifocal or circuit-level modulation with pattern changes across trials or subjects	Dynamic patterned field	High spatial degrees of freedom, reprogrammability, adaptive energy control	Phased array	Complexity and cost rise with channel count and calibration demands
Longitudinal study prioritizing session-to-session reproducibility	Mostly fixed	Reproducible placement, individualized planning, explicit safety and reporting workflow	Hologram/lens-assisted system if geometry can be reproduced; phased array if adaptive per-session correction is needed	Reproducibility depends on how tightly positioning and coupling can be controlled
Exploratory target-mapping or protocol-development study	Dynamic	Broad usable steering range, rapid iteration, adaptable correction across candidate sites	Phased array	Highest implementation burden

Entries refer to relative suitability, not absolute superiority. Realized focal quality, positioning robustness, and safety depend strongly on frequency, aperture, focal depth, skull path, correction method, coupling, and planning workflow.

In summary, subdividing an aperture into multiple elements does not by itself reduce the diffraction-limited focal volume when aperture, transmit frequency, focal depth, effective source geometry, and aberration correction are matched (O’Neil, 1949; Kossoff, 1979; Peralta et al., 2020). However, this statement should not be interpreted to mean that phased arrays and single-element transducers are functionally equivalent in transcranial applications. Skull-induced aberrations can broaden, displace, or split the focus, and phased arrays provide a powerful means of dynamically correcting these effects. Single-element systems can also achieve patient-specific correction when paired with appropriately designed acoustic holograms or lenses. The most useful comparison is therefore not whether multi-element arrays are categorically more precise, but which architecture best balances focal quality, steering requirements, aberration correction, cost, complexity, and scalability for a given application.

## Data Availability

The original contributions presented in the study are included in the article/supplementary material, further inquiries can be directed to the corresponding author.
